# The Influence of Graphene Addition on the Properties of Composite rGO/PAN Membranes and Their Potential Application for Water Disinfection

**DOI:** 10.3390/membranes10040058

**Published:** 2020-03-29

**Authors:** Beata Fryczkowska, Alicja Machnicka, Dorota Biniaś, Czesław Ślusarczyk, Janusz Fabia

**Affiliations:** 1 Institute of Environmental Protection and Engineering, Faculty of Materials, Civil and Environmental Engineering, University of Bielsko-Biala, Willowa 2, 43-309 Bielsko-Biala, Poland; amachnicka@ath.bielsko.pl; 2 Institute of Textile Engineering and Polymer Materials, Faculty of Materials, Civil and Environmental Engineering, University of Bielsko-Biala, Willowa 2, 43-309 Bielsko-Biala, Poland; dbinias@ath.bielsko.pl (D.B.); cslusarczyk@ath.bielsko.pl (C.Ś.); jfabia@ath.bielsko.pl (J.F.)

**Keywords:** composite membranes, graphene, polyacrylonitrile, nanoparticles, disinfection

## Abstract

The paper presents a method of obtaining composite polyacrylonitrile-based (PAN) membranes with the addition of reduced graphene oxide (rGO). The membranes were obtained using phase inversion method from a homogeneous rGO dispersion in a solution of PAN dissolved in N, N-dimethylformamide (DMF). The impact of the amount of rGO addition to the PAN matrix on the physicochemical, structural, transport, and separation properties and on fouling resistance was studied. Composite membranes, due to the method of preparation used and the addition of rGO, are characterized by very good transport properties (~390 L/m^2^ h) and by a high degree of protein retention (85%). Reduced graphene oxide has biocidal properties, which, as we have shown, depend on the size of nanoparticles and the type of microorganism. rGO/PAN membranes, on the other hand, show biostatic properties against Gram-negative bacteria (*Escherichia coli*), Gram-positive bacteria (*Staphylococcuc aureus*) and fungi (*Candida albicans*). Thus, the obtained composite membranes can be potentially used in water disinfection.

## 1. Introduction

The demand for water is growing at an enormous pace, since there are almost 7 billion people in the world. More than a billion people do not have access to potable water, and 2.6 billion people live in poor sanitation conditions. A large part of the potable water is not fit for direct consumption, and it needs to undergo purification processes. An important method of water purification is disinfection, which aims to destroy live and spore forms of pathogenic organisms and to prevent their secondary development, e.g., in a water supply system. Microorganisms present in water are partly removed together with suspensions in sedimentation and filtration processes. The complete removal of microorganisms requires special measures used in chemical and physical methods. The commonly used chemical disinfectants for water include: chlorine, ozone, and chloramines [1]. Physical methods of water disinfection include: boiling, pasteurization and ultraviolet exposure [2], the use of ultrasound or gamma radiation, as well as ultrafiltration [3]. Ultrafiltration is one of pressure membrane processes that employ various types of polymeric membranes.

Polyacrylonitrile (PAN) is a polymer widely used in membrane processes, among others due to its excellent chemical stability and mechanical strength [4]. Zhang et al. obtained PAN nanofiber mats for cleaning aerosols of microorganisms (*Escherichia coli* vegetative cells and *Bacillus subtilis* endospores) [5]. PAN/polysulfone composite membranes were used to purify ground and surface waters of *Escherichia coli* bacteria [6]. Polyacrylonitrile can also be easily physically modified, e.g., by functionalizing its surface or adding an additive to the PAN matrix. Nanofiber membranes for biomedical filtration devices were described by Kharaghani et al. who received them of PAN functionalized with silver nanoparticles [7]. The nanofilters they presented had bactericidal effect against *Escherichia coli* and *Staphylococcus aureus*. Bactericidal composite membranes based on the PAN matrix with the addition of silver and *β*-cyclodextrin were obtained by electrospinning [8]. Youan et al. developed a composite fabric based on PAN and polymer carbon nitride for photocatalytic water disinfection and removal of *Acinetobacter baumannii*, *Escherichia coli,* and *Staphylococcus aureus* [9]. Other researchers received the silver nanowires polyacrylonitrile/thermoplastic polyurethane composite membrane, which can be used in electrochemical water disinfection [10].

Membranes that are used in water disinfection processes must not be biofouling. To prevent this, physical or chemical modification of such membranes should be performed. PAN is a polymer that can be easily modified physically, e.g., by introducing an additive. Chitosan coated iron oxide nanoparticles were impregnated into PAN based hollow fiber membrane by the team of Mukherjee et al. [11], and the resulting composite demonstrated strong bactericidal properties.

**Figure 1:**
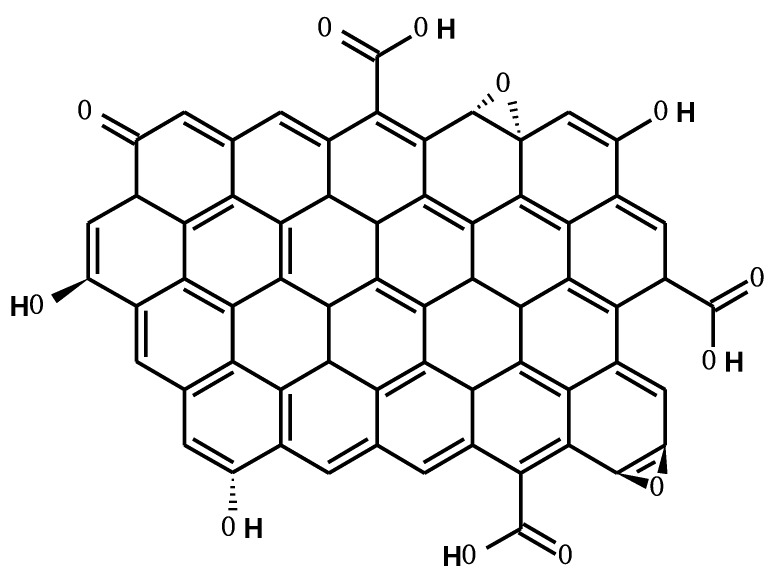
Chemical structure of reduced graphene oxide (rGO).

An interesting addition to the PAN may be graphene, which is a two-dimensional material. Depending on the method of preparation, it can be built of one or several graphene layers, which affects its potential application. Chemical or thermal reduction of graphene oxide (GO) results in obtaining reduced graphene oxide (rGO), which, among others, has bactericidal properties [12,13,14,15]. The rGO sheets are heavily oxygenated with basal planes containing hydroxyl and epoxide functional groups, in addition to carbonyl and carboxyl groups located at the sheet edges (Figure 1). This makes rGO strongly hydrophilic, allowing easy swelling and dispersing of graphite oxide in water [16]. According to the literature, a team of researchers functionalized graphene flakes, which were then used to obtain a graphene–poly(acrylonitrile-co-maleimide) composite with hydrophilic properties [17]. A complicated synthesis of the rGO/PAN composite was conducted by Mo et al. [18]. At first, they functionalized graphene oxide (GO) with siloxanes containing amine groups, and then reduced it and copolymerized with acrylonitrile and acrylamide. The team of Ruiz et al. obtained graphene quantum dots/polyacrylonitrile membranes by electrospinning [19].

Prusty et al. described the possibility of obtaining a rGO/PAN composite as a result of emulsion polymerization of acrylonitrile in the presence of rGO water dispersion [16]. In our publication, we present a simple method of obtaining rGO/PAN composite membranes. At the beginning, reduced rGO was obtained by the GO thermal reduction method. Then, rGO was dispersed in DMF, then the PAN was dissolved in the obtained dispersion, resulting in membrane-forming solutions with a very wide range of reduced graphene oxide concentrations (0.11% to 29.4% *w*/*w*). RGO/PAN membranes were obtained using phase inversion method by coagulation in water followed by examination of their physicochemical and transport properties. In our work, the separation properties of rGO/PAN membranes for protein solution—bovine serum albumin (BSA)—were also examined, checking whether the addition of rGO results in composite membranes fouling resistance properties, so characteristic of membranes from pure PAN) [20]. Microbiological tests conducted on *Escherichia coli (E. coli)*, *Staphylococcuc aureus (S. aureus)* and *Candida albicans (C. Albicans)* have shown that the membranes built on the PAN matrix with the addition of rGO have biostatic properties, which makes them potentially useful in water disinfection processes.

## 2. Experimental

### 2.1. Materials

PAN (MW = 85,000)—copolymer (93.9 % acrylonitrile/5.8 % methyl acrylate/0.3 % methallyl sulfonate) was obtained from Goodfellow Cambridge Ltd, Huntingdon, England. Graphite powder (<20 μm and <150 µm), bovine serum albumin (BSA) (Mw = ~66 kDa) were purchased from Sigma-Aldrich, Poznań, Poland. NaNO_3_, min. 95 % H_2_SO_4_, KMnO_4_, 30 % H_2_O_2_, NaCl, DMF were purchased from Avantor Performance Materials Poland S.A (Gliwice, Poland). The *S. aureus* (ATCC 33741-B1), *E. coli* (ATCC 35925-B2), and *C. albicans* (ATCC BAA-473) were purchased from ATCC (American Type Culture Collection, Gaithersburg, MD, USA). Blood agar, Chapman medium, MacConkey medium, Candida agar were purchased from BTL Ltd (Department of enzymes and peptones, Łódź, Poland).

### 2.2. Synthesizing Graphene Oxide (GO) and Reduced Graphene Oxide (rGO)

GO was obtained using the modified Hummers method [21]. Two grams of graphite powder were added to 46 cm^3^ of H_2_SO_4_ and the resulting suspension was stirred for 30 min in an ice bath. Then, 6 g of KMnO_4_ was slowly added to the solution, in such a way to prevent exceeding a temperature of 20 °C. The contents of the beaker were warmed to 35 °C and stirred for 2 h. Next, 92 cm^3^ of distilled water was added. To remove the remaining KMnO_4_, 80 cm^3^ of warm distilled water (60 °C) together with 50 cm^3^ of a 3% aqueous solution of H_2_O_2_ were added. The resulting sample was centrifuged and repeatedly rinsed with distilled water until pH of 7 was achieved. The GO prepared in this way was thermally reduced. The low-temperature thermal reduction underwent in nitrogen atmosphere at a temperature below 300 °C with a heating rate of 30 °C/min. After the reduction, the yellow-brown GO sample resulted in a black rGO powder.

The reaction of GO synthesis and thermal reduction to rGO was performed for two types of graphite with particle sizes of: <20 µm (rGO20) and <150 µm (rGO150).

### 2.3. Forming PAN Membranes

PAN membranes were obtained by phase inversion method. Twelve percent *w*/*w* PAN solution in DMF was initially prepared. The polymer was dissolved at 50 °C and then cooled down to room temperature. The PAN solution was poured onto a clean glass plate and spread using an applicator with a gap width of 0.2 mm [22]. Finally, the polymer film was rapidly coagulated in distilled water at room temperature until the membrane detached from the glass. The precipitated membranes (membrane “0”) were dried in air by interposing a layer of tissue paper. To prevent wrinkling of the the membrane, a glass plate load was used [23].

### 2.4. Forming GO/PAN Membranes

Membrane-forming solutions containing 12% *w*/*w* of PAN in rGO/DMF solution were prepared. For this purpose, the appropriate amounts of rGO (rGO20 and rGO150) and DMF were weighed and mixed thoroughly. Then the appropriate amounts of PAN were added and mixed at 50 °C until the polymer dissolved (Table 1). The rGO/PAN/DMF solution prepared this way was then poured onto a clean glass plate and spread using a casting knife with an adjustable thickness fixed at 0.2 mm. Finally, they were rapidly coagulated in distilled water at room temperature until the membrane detached from the glass. The precipitated membranes were air-dried by interposing a layer of tissue paper, and then loading with the glass plate.

During the research, it turned out that due to the large particle size of rGO (<150 µm) it is impossible to prepare a homogeneous solution of rGO150/polyacrylonitrile-based (PAN)/N,N-dimethylformamide (DMF). In addition, composite membranes obtained from this type of graphene were characterized by a large number of holes throughout their entire surface, which would hinder testing of transport and separation properties. Therefore, reduced graphene oxide obtained from graphite with a size <20 µm was used to study the preparation of rGO/PAN membranes.

### 2.5. General Characterization

Elmetron MG-1 thickness gauge was used for thickness (*l*) measurement of the membranes. Samples of 1 × 1 cm were weighed using a Sartorius CP224S-0CE analytical balance with an accuracy of 0.0001 g.

The mass per unit area (*W_s_*) [g/cm^2^] and the apparent density (*d_m_*) [g/cm^3^] of the membranes were calculated using the following formulas (1) and (2):

(1) $$W_{s}=\frac{w}{s}$$

(2) $$d_{m}=\frac{w}{s\times l}\;$$

where: *w*—mass of a membrane with an area of 1 cm^2^, *s—*membrane surface area [cm^2^], *l—*membrane thickness [cm].

The sorption of water (*U*) was measured in the following way: dry membrane samples (*W_d_*) with dimensions of 1 x 1 cm were weighed on an analytical balance with an accuracy of 0.0001 g and then immersed in distilled water for 10 seconds. The membranes were then blotted on filter paper and weighed again when wet (*W_w_*). The water sorption was calculated according to the following formula (3):

(3) $$U=\frac{W_{w}-W_{d}}{W_{d}}\times 100\%$$

The porosity of the membranes (*ε*), defined as the ratio of pore volume to the volume of the membrane, was calculated using formula (4) [24]:

(4) $$\varepsilon =\frac{\left(W_{w}-W_{d}\right)/d_{w}}{\left(W_{w}-W_{d}\right)/d_{w}+W_{d}/d_{p}}\times 100\%$$

where *d_w_*—density of distilled water (0.998 g/cm^3^) and *d_p_*—polymer density (1.184 g/cm^3^) [25].

The static contact angle measurement was performed using a goniometer (FIBRO System AB PG-1, Testing Machines, Inc., New Castle, DE, USA); therefore, the tests were restricted to the skin (top) layer of the membranes.

### 2.6. Transport Properties

The transport properties of the formed membranes were tested using a Millipore Amicon 8400 ultrafiltration cell with a 350 mL capacity and a 7.6 cm membrane diameter, equipped with an equalizing tank of 800 mL. Initially, dry membranes were immersed in distilled water for 1 h. Next, they were treated with distilled water for 2 more h under a pressure of 0.2 MPa to improve their stability. UF tests were performed at the pressures of 0.1, 0.15, or 0.2 MPa. Tests for the next operating pressure were carried out until the flow through the membrane stabilized. Permeate flux (*J_v_*) was calculated using formula (5):

(5) $$J_{v}=\frac{Q}{A\times t}$$

where *J_v_* is water flux [L/m^2^h], *Q* is the permeate volume (L), *A* is the effective membrane area [m^2^], and *t* is the permeation time [h].

### 2.7. Rejection Measurements

Studies of membrane transport properties using anhydrous aqueous solution of BSA at a concentration of 0.1 g/L were also performed. Three hundred milliliters of the BSA solution was poured into the ultrafiltration cell with a previously tested membrane. The permeation process was carried out at a working pressure of 0.2 MPa and 30 mL doses of permeate were tapped, simultaneously measuring the time of the permeate discharge from the test tank. The tests were performed ten times for each membrane. Volumetric permeate flux (J_v_) was calculated using formula (5), assuming that in this case Q is the permeate volume (BSA solution).

The concentration of BSA in the permeate was determined using UV-Vis spectrophotometry (Perkin Elmer Lambda 35 UV-Vis spectrophotometer) by measuring the absorbance at 280 nm. Based on the calibration curve, the BSA concentrations in each sample were calculated. Then, using formula (6), the BSA rejection performance (*R*), closely associated with the occurrence of fouling, was calculated.

(6) $$R=\left(1-\frac{C_{p}}{C_{f}}\right)\times 100\%$$

where *R*—rejection performance of the membrane [%] and *C_p_* and *C_f_*—concentrations of BSA in the permeate and feed solution [g/L], respectively.

### 2.8. Microbiological Analysis

The samples were exposed to bacteria and fungi capable of causing infections in humans, i.e., the Gram-positive *S. aureus* and Gram-negative *E. coli*, and *C. albicans.* The microorganisms were grown on blood agar. Then they were incubated at 36 ± 2 °C for 24 h. Obtained cultures were washed out with 1 ml of physiological salt solution, and 0.1 mL was added to the sterile selective agar. The following mediums were used in the cultivation of the microorganisms: Chapman agar—*S. aureus*, MacConkey agar—*E.coli* and Candida agar—*C. albicans*. Seeding of "grated tiles" was employed. Samples were placed in the centre of the Petri plate. The Petri plates with samples were then put into a laboratory heater and kept there at 36 ± 2 °C for 24 h. At the beginning, sterile paper discs (diameter 1.0 cm) were impregnated with two drops of rGO solution (concentration was: 0.001; 0.01; 0.1 g/L). Five tests were performed for each type (rGO20 and rGO 150) and each rGO concentration. Subsequently, control samples of PAN membranes (membrane 0) and rGO/PAN composite membranes testing samples (membrane A–H) were prepared. The membranes sizes were 1 × 1 cm. Five tests were performed for each type of membrane. Growth inhibition zones were analyzed using stereoscopic microscope with Olympus CCD ARTCAM camera.

### 2.9. Analytical Methods

All measurements were performed using a Nicolet 6700 FT-IR spectrometer (Thermo Electron Corp., Madison, WI, USA) equipped with photoacoustic MTEC model 300 accessory. Photoacoustic testing samples were placed in a special snap holder. The measurement parameters used were as follows: resolution, 4 cm^−1^; spectral range, 500–4000 cm^−1^; (DTGS) detector; number of scans, 64. Data was collected and post-processed using OMNIC software (v. 8.0, Thermo Electron Corp.).

The X-ray investigations were carried out with a diffractometer model URD-65 (Seifert, Dresden, Germany). CuKα radiation was used at 40 kV and 30 mA. Monochromatization of the beam was obtained by means of a nickel filter and a graphite crystal monochromator placed in the diffracted beam path. A scintillation counter was used as a detector. Investigations were performed in the range of the angle 4° to 60° in steps of 0.1°. To quantitatively examine the crystallinity of the membranes studied their crystallinity index was evaluated from WAXS measurements. For this purpose each X-ray diffractogram was deconvoluted into crystalline and amorphous scattering components using the profile fitting program WaxsFit [26]. Each peak was modeled using a Gaussian-Cauchy peak shape. The crystallinity index was calculated as a ratio of the area under crystalline peaks to the total area of the scattering curve.

Thermal studies of the membranes were conducted using a TA Instruments MDSC 2920 differential scanning calorimeter (TA Instruments, New Castle, DE, USA). The DSC curves obtained were analyzed using the TA Instruments Universal V4.5A software package. Measurements were performed under a nitrogen atmosphere (flow rate 40 mL/min) while heating at 10 °C/min and 60 °C/min from −40 to 320 °C.

Surface and cross-sections morphology of membranes were analyzed using Phenom ProX scanning electron microscope (SEM) by Thermo Fisher Scientific (Pik Instruments, Piaseczno, Poland), operating at 10 kV, equipped with an energy-dispersive spectrometer (EDS). Liquid nitrogen was used to prepare the cross-sections of membranes, in which the samples were frozen and then broken and sprayed with a 10 nm thick layer of gold using diffusion method, by LEICA ACE 200 low vacuum sprayer (Pik Instruments, Piaseczno, Poland).

## 3. Results and Discussion

### 3.1. Characteristics of rGO

As a result of low-temperature thermal reduction, rGO was obtained. The elemental composition, determined by means of the EDS probe built into SEM, for individual rGO varieties was 81.5% at. C and 18.5% at O (rGO20); 79.2 % at. C and 20.8% at O (rGO150), respectively. Based on the WAXS curves, the rGO particle size, the number of graphene layers and the interplanar distance were calculated. The rGO particle size was <20 µm (for rGO20) and 150 µm (for rGO150). The average number of rGO layers was 4, while the distance between subsequent graphene layers was 0.37 nm for rGO20 and 0.38 nm for rGO150.

### 3.2. Membrane Characteristics

**Figure 2:**
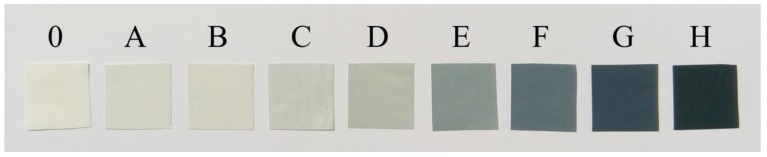
Images of membrane pure PAN membrane (membrane 0) and rGO/PAN composite membranes (membranes A-H).

The membranes obtained in the experiment differed in color depending on the amount of rGO addition to the PAN matrix (Figure 2). Membranes from pure PAN (0) were white. On the other hand rGO/PAN (A–H) membranes were characterized by different shades of grey. The more rGO added in the composite membrane, the darker the color. Black is dominant in the H membrane, which contains the highest addition of rGO (29.4% *w*/*w*).

The external appearance of the membranes (presented in Figure 2) does not say much about the morphology of their structure, so scanning electron microscopy (SEM) pictures were taken. The use of SEM allowed to examine the surface and cross sections morphology of produced composite rGO/PAN membranes and the pure PAN membranes.

**Figure 3:**
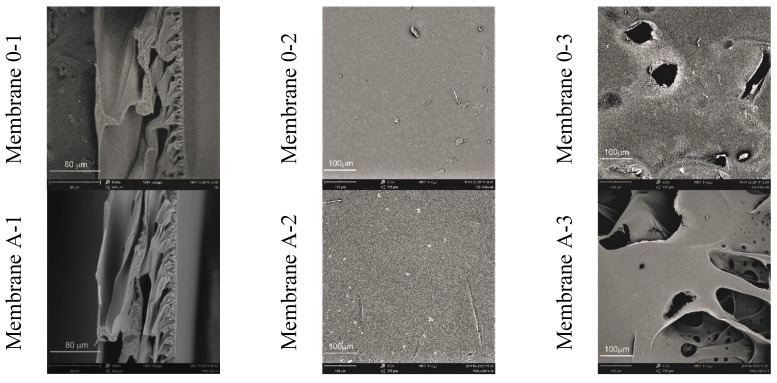

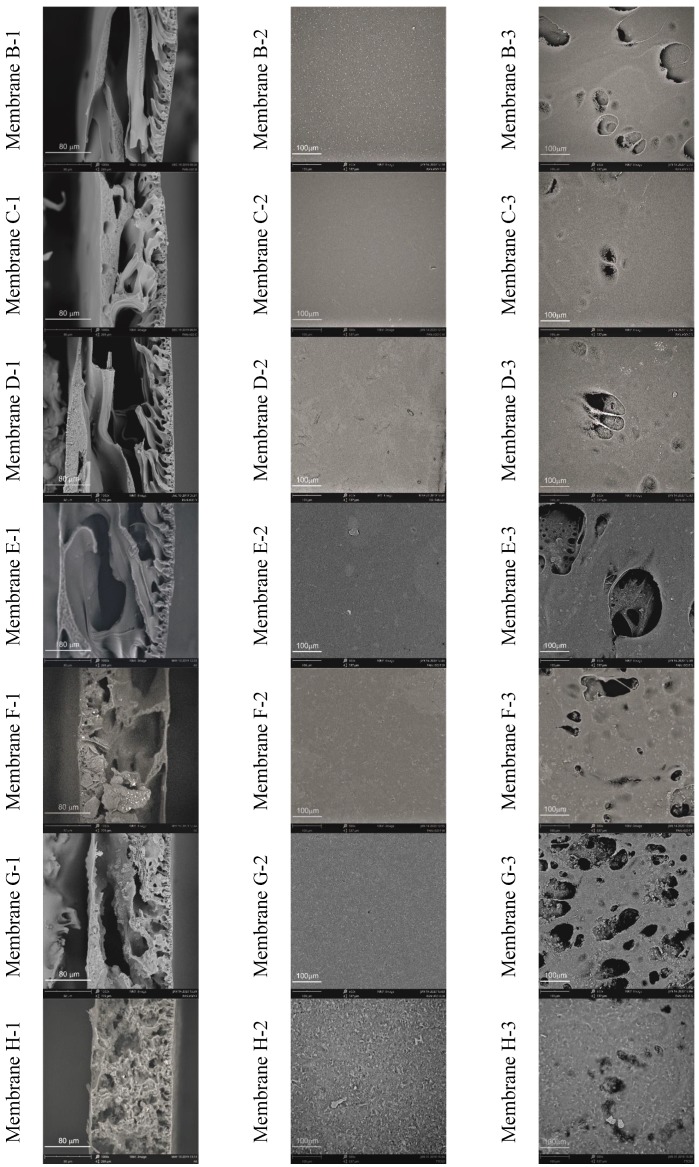
Images (SEM) of pure PAN and rGO/PAN composite membranes: (**1**) Cross-section; (**2**) top (skin) layer; (**3**) bottom layer.

Figure 3 (1) confirms the asymmetric construction of all membranes. For membrane 0 and A to G a dense skin layer with a thickness of 3.5–6.0 mm is clearly visible. The layer is hardly visible only in the case of membrane H, which contains almost 30% *w*/*w* of the rGO addition. The cross-sectional appearance is similar for most obtained membranes. Looking from the skin side, the membranes are covered with small capillaries, which become larger and thicker as they move away from the skin [27]. On the other hand, the support layer, is made of large, porous chambers and makes up the majority of the membrane surface. Analyzing in more detail the cross-sectional images of membranes D and E, it can be observed that the walls of the support layer show numerous lumps with a size of several µm. On the other hand, the SEM images of the cross-sections of membranes F, G, and H show randomly scattered flakes—perhaps rGO and its agglomerates, as indicated by their dimensions above 20 µm.

Analyzing the images of the surface of membranes from the skin layer (Figure 3 (2)) one can observe a smooth and even surface morphology, which changes for individual membranes. Membranes A and B show small but visible bright spots, which may indicate fragments of rGO protruding from the membrane, whose surface is not flat, but folded [28]. SEM images of membranes D, E, F reveal inclusions that transform into microcracks for subsequent membranes (G and H). The structure of the support layer (Figure 3 (3)) of the membranes obtained in the experiment is even more interesting to observe. There are random holes and pores on it, and, at higher magnifications, a very interesting and rich multidimensional structure is revealed.

The paper analyzes the way in which the amount of rGO additive added to the PAN matrix influences the physicochemical properties of rGO/PAN composite membranes. Tests of physicochemical properties were carried out, which allowed to characterize membranes from pure PAN as well as rGO/PAN membranes. The obtained results are summarized in Table 2.

Analysing the results of thickness measurements (Table 2), it can be noticed that the thickness of the membrane 0 is ~190 µm. The introduction of a small amount of rGO into the PAN matrix caused the thickness of membrane A to increase by less than 9 µm, which can also be seen in the scanning microscope images (Figure 3). It can therefore be concluded that an addition of 0.11% *w*/*w* of rGO slightly slows down the membrane coagulation process, which increases its thickness. For other membranes, a slow decrease in their thickness is observed with an increase in the amount of rGO in the direction from membrane B to H. Thus, the introduction of reduced graphene oxide to the PAN matrix in an amount above 0.11% *w*/*w* results in a slight acceleration of the coagulation process of composite rGO/PAN membranes in water.

The results of mass per unit area measurements (Table 2) indicate a decrease in the mass of composite membranes in the direction from membrane A to E, compared to membrane 0. The addition of reduced graphene oxide facilitates coagulation of composite membranes. At the same time, it facilitates water penetration, which ultimately leads to a decrease in the mass per unit area of rGO/PAN membranes. However, for membranes that contain high amounts of rGO, namely: 7.7% *w*/*w* (membrane F), 14.3% *w*/*w* (membrane G), 29.4% *w*/*w* (membrane H) an increase in mass per unit area is observed. The obtained results may prove that a large part of the mass of these composite membranes is rGO itself.

Analyzing the apparent density values for all membranes obtained (Table 2), it can be seen that the pure PAN membrane has a density of 0.170 g/cm^3^. In the case of rGO/PAN membranes, it can be noticed that with the addition of rGO to the polymer matrix (membranes: A, B, C, D), the density of membranes drops to 0.140 g/cm^3^ for membrane D. This is due to their low mass per unit area and high porosity. The density of membranes E, F, G, and H, on the other hand, increases with the increase in rGO content in membranes.

Water sorption tests (Table 2) indicate that the membranes obtained in the experiment were characterized by water sorption values at the level of 209–330%. For membrane 0, water sorption is ~250% and porosity is 74.5%. Analyzing the results obtained for membranes A, B, C, D, and E, an increase in water sorption is observed, up to a value of 329.8% for membrane E. Moreover, as the rGO content in rGO/PAN membranes increases, their porosity also increases in the following order: 74.6%, 75.1%, 76.9%, 77.0%, and 79.4% for membranes: A, B, C, D, and E, respectively. However, the introduction of rGO addition above 4% *w*/*w* results in a decrease in sorption properties of composite membranes F, G, and H. The water sorption determined for the H membrane is ~209%, which is 40% less compared to a membrane obtained from pure PAN. At the same time, the porosity of these membranes is reduced to 75.3%. The observed decrease in water sorption, accompanied by a decrease in the porosity, of membranes F, G, and H may be the result of accelerating the coagulation process of these membranes in water.

Analyzing the contact angle values (Table 2) of membrane 0 (52°) and composite membranes A–H (49.5–40.7°), it can be concluded that the addition of rGO reduces these values, regardless of its amount in the PAN matrix. Consequently, rGO slightly improves the hydrophilic properties of membranes [29].

The comparison of the results of testing the physicochemical properties of composite membranes leads to a conclusion that a small addition of rGO to the PAN matrix (up to 4% *w*/*w*) reduces membrane thickness, mass per surface area, apparent density and contact angle, while increasing water porosity and sorption. On the other hand, the introduction of subsequent portions of rGO (above 4% *w*/*w*) into the PAN matrix results in an increase in their density and contact angle, while the porosity and sorption properties of rGO/PAN composite membranes decrease.

### 3.3. Transport and Separation Properties of the Membranes

**Figure 4:**
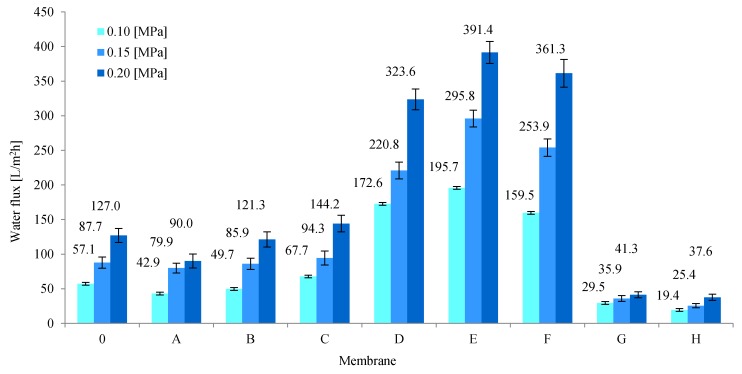
Values of volumetric permeate flux for membranes made from pure PAN (membrane 0) and composite rGO/PAN membranes (membranes A–H).

An important parameter determining the transport properties of membranes is the volumetric permeate flux (Figure 4). Pure PAN membranes (membrane 0) obtained in the experiment were characterized by permeate flux with subsequent values: 51.5, 87.7, and 127.0 L/m^2^h for operating pressures of distilled water: 0.1, 0.15, and 0.2 MPa. On the other hand, composite membranes A and B, containing a small amount of rGO as an additive to the PAN matrix, were characterized by lower values of the volumetric permeate flux. The obtained results may indicate the formation of connections between the functional groups of the polymer and oxygen groups on the edges of the flakes of reduced graphene, which results in a more compact structure of the skin layer of the obtained rGO/PAN membranes, as also confirmed by SEM images (Figure 3 A-2 and B-2). Further increase in the rGO concentration in composite rGO/PAN membranes causes an increase in the specific permeate flux. The highest values of the volumetric permeate flux were found in membranes D, E, F which contained 0.83%, 4.9%, 7.7% of rGO additive, respectively. The values were 323.6, 391.4, 361.3 L/m^2^h, respectively for an operating pressure of 0.2 MPa. Such a large increase in flow through the membranes may be caused by the high content of rGO in the PAN matrix, which results in agglomeration of nanoparticles in the skin layer, which can also be seen in SEM images (Figure 3 D-2, E-2, F-2). This also results in a decrease in the contact angle values for membranes D, E and F (Table 2). For membranes that contain high amounts of rGO (membranes G and H), the volumetric permeate flux drops dramatically to 41.3 and 37.6 L/m^2^h for a pressure of 0.2 MPa. The observed phenomenon may be the result of reduced porosity, and thus water sorption (Table 2), which are the result of a change in the internal structure of the membranes, visible in SEM images (Figure 3 G-1, H-1).

**Figure 5:**
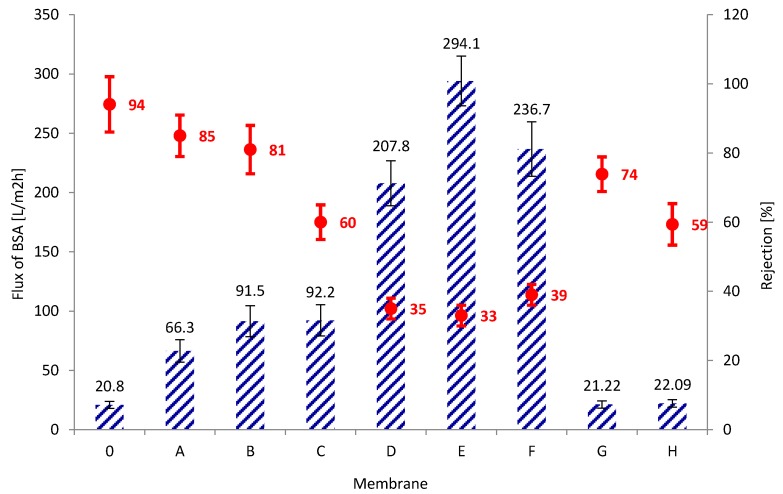
The volumetric permeate flux and rejection coefficients for BSA determined for subsequent membranes at a working pressure of 0.2 MPa.

Study of transport properties was extended to include tests of separation properties against BSA (Figure 5). The obtained results indicate that under the influence of BSA there is a decrease in the volumetric permeate flux for all tested membranes. The largest decrease was ~84% and it was recorded for membrane 0. For this membrane, the rejection is also high and amounts to 94%, which is consistent with the lack of resistance of pure PAN to fouling [20]. Very good rejection results were also determined for membranes A and B, which contained 0.11% and 0.22% *w*/*w*, respectively, of rGO in the PAN matrix. They were 85 and 81%, with the volumetric permeate flux decreasing only by ~26% for membrane A and by ~25% for membrane B. Membranes D, E and F, which were characterized by the highest values of volumetric permeate flux for distilled water, under the influence of BSA reduce flux values by ~36% (for membrane D); ~25% (for membrane E); ~ 35% (for membrane F). For these membranes, at the same time, an exceptionally low rejection values (in the range of 33–39%) were recorded, which indicates that membranes D, F, and G have pores in the skin layer large enough to allow free penetration by BSA. The obtained results confirm the physicochemical properties, which indicated that the membranes are relatively thin (170–183 µm), with low apparent density (140–149 g/dm^3^) and high water sorption (325–330%) (Table 2).

The obtained results of the separation properties of rGO/PAN composite membranes have shown that a small addition of rGO results in good separation of protein molecules without significantly reducing the liquid flow through the membrane. rGO nanoparticles can form links with PAN functional groups, lowering the contact angle (Table 2), resulting in repelling BSA particles and preventing fouling. A feature of a protein dissolved in water is the ability to form tightly packed structures with unpolished core and polar functional groups on the outside [30], which in our case prevents clogging of membranes A and B. On the other hand, the introduction of rGO in an amount of 0.45% *w*/*w* and more (D, E, F, G, and H) adversely reduces the BSA rejection value on the composite membranes, which prevents their use with the protein-like chemical compounds.

### 3.4. Biocidal Properties of rGO Solutions and Biostatic Properties of rGO/PAN Membranes

**Figure 6:**
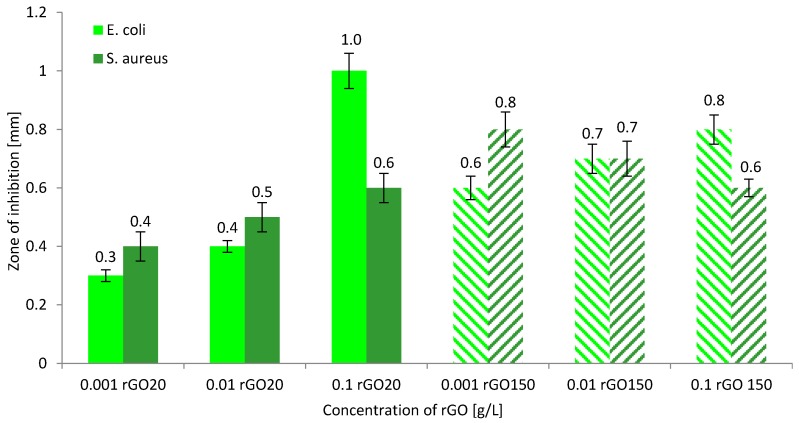
Growth inhibition zones for *E. coli* and *S. aureus* bacteria depending on the concentration of reduced graphene oxide (rGO20 and rGO150).

The literature reports that thermally reduced graphene oxide has bacteriostatic and bactericidal properties [3,31,32,33]. The destruction of bacterial cells occurs as a result of physical and chemical interactions with rGO. One of the reasons for bacteria destruction may be breaking of the thin cell membrane, resulting in a leakage of cytoplasm, described in the literature [14,15]. In another case, bacteria death occurs as a result of wrapping and/or trapping the bacteria, resulting in membrane stress and/or oxidative stress, as well as loss of cell viability and DNA fragmentation [15,34]. Therefore, we conducted tests with the use of thermally reduced graphene oxide with two different nanoparticle sizes. The research indicated that both rGO20 and rGO150 nanoparticles show biocidal properties and inhibit the multiplication of tested bacteria (Figure 6). In the case of Gram-negative bacteria, it was observed that as the concentration of rGO increases, so does the area of inhibition, which for rGO20 is 1 mm, while for rGO150 it is 0.8 mm. However, for Gram-positive bacteria it is observed that for small rGO flakes the inhibition zone is 0.6 mm (for rGO20 concentration of 0.1 g/L), and for large rGO flakes it decreases to this value as the rGO150 concentration increases. In addition, the difference in rGO biocidal properties is closely related to the structure of bacterial cells [31]. *E. coli* are rod-shaped, with dimensions 2 × 0.8 µm and a thin cell membrane. *S. aureus,* on the other hand, have a spherical structure, with dimensions of 1 × 0.8 µm, surrounded by a thick cell membrane. A slight difference in the biocidal properties of rGO may be related to the fact that at the same weight concentration of nanoparticles, the probability of *E. coli* bacteria meeting rGO150 flakes is lower than for rGO20 flakes. In addition, agglomeration of large rGO particles is likely in solutions with a higher concentration (0.1 g/L), which in the case of bacteria of a spherical shape (*S. aureus*) hinders its destruction, which is manifested by the inhibition zone reduced to 0.6 mm.

**Figure 7:**
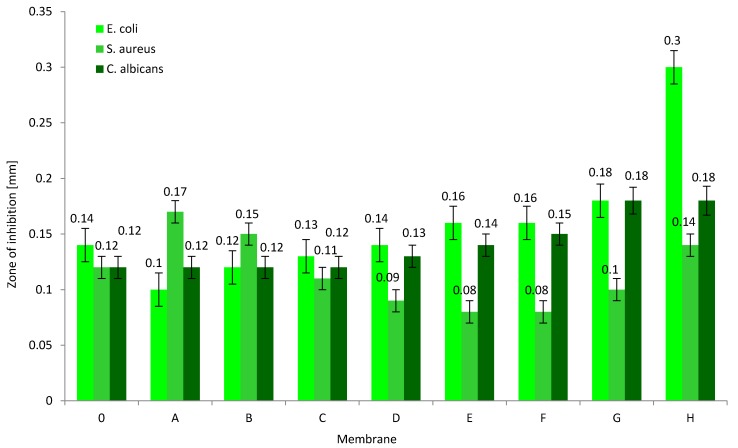
Zones of microorganism growth inhibition in the presence of pure PAN membranes (membrane 0) and rGO/PAN membranes (membranes A–H).

The aim of our research was to obtain composite membranes with biocidal properties that could be used in water disinfection processes. In the literature, there are many examples of polymer composites containing an rGO addition that have biocidal properties [14,35]. Therefore, we conducted microbiological tests on rGO/PAN composite membranes obtained in the experiment. The following microorganisms were selected: *S. aureus*, *E. coli*, and *C. albicans*, and the obtained results are summarized in Figure 7. These studies show that membranes "0" show biostatic properties, which has already been described in the literature [6]. Pure PAN causes the occurrence of narrow inhibition zones with a width of 0.12–0.14 mm (Figure 7). However, for composite membranes rGO/PAN it is observed that the addition of rGO of up to 0.83% *w*/*w* does not increase the *E. coli* growth inhibition zone. Only larger amounts of nanoparticles in the polymer matrix effectively interact with thin Gram-negative bacteria membranes causing their death within 3 mm of the membrane H sample. A similar nature of the changes was observed for *C. albicans* fungi. The fungal colonies growth inhibition zones slowly increased from a width of 0.13 mm (membrane D) to 0.18 mm (membrane H). On the other hand, when analyzing the results of biological activity of rGO/PAN membranes (Figure 7) against *S. aureus* bacteria, it can be observed that only a small addition of nanoparticles (0.11–0.22% *w*/*w* of rGO) to the PAN matrix causes an increase in the bacterial growth inhibition zone. Comparing the results obtained in the research of Zou et al. [15] it can be concluded that the surface of the membrane from the skin side is covered with sharp fragments of rGO, which easily destroy *E. coli*, rupturing their cell membrane. Such structure of rGO/PAN membranes also prevents the growth of *S. aureus*, but to a lesser extent. Therefore, it can be concluded from the research that the rGO/PAN membranes obtained in the experiment show biostatic properties, which, combined with the separation properties, create the potential for their use in water disinfection processes.

### 3.5. Structural Studies

#### 3.5.1. FTIR

**Figure 8:**
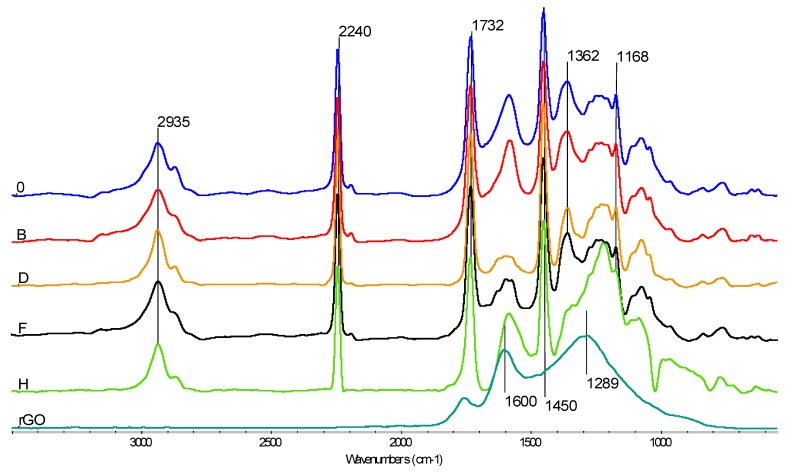
FTIR spectra of the composite rGO/PAN membranes (**B**, **D**, **F**, **H**), the pure PAN membrane and the pure rGO.

In FTIR spectra (Figure 8), characteristic absorption bands can be observed via the oscillators in PAN: 2935 cm^–1^ are C–H oscillators stretching vibrations, 2240 cm^–1^ are C≡N oscillator stretching vibrations, while 1450 cm^–1^ and 1362 cm^–1^ are the deformation vibrations in CH_2_ and CH groups [16]. The 1600 cm^–1^ band is characteristic for C=C stretching bonds, occurring in rGO, as well as in rGO/PAN membranes, where its intensity increases with the increase in the amount of the additive [29]. The band of approximately 1732 cm^–1^ occurring in the spectra is characteristic for the C=O oscillator stretching vibrations in ester moieties and may come from raw PAN (which is a terpolymer) and from rGO. The absorption peak at 1168 cm^–1^ comes from the C–O binding [27,29], originating from the PAN, and occurring in membrane 0 and rGO/PAN membranes. And the band at 1289 cm^−1^ appearing in the rGO spectrum comes from the stretching vibrations of the CO oscillator in the ether moiety [36].

#### 3.5.2. WAXS

**Figure 9:**
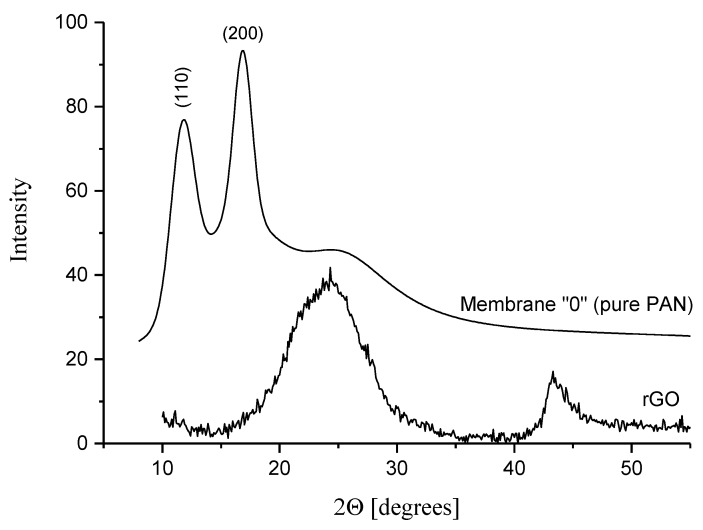
X-ray diffractograms of Membrane 0 (pure PAN) and the pure rGO (for better visualization, the diffraction curves have been shifted along the ordinate axis).

The morphology of all membranes was investigated with X-ray diffraction. The membrane “0” (pure PAN) exhibits two main diffraction peaks at 2θ = 11.9° and at 2θ = 16.9° which are characteristic for the paracrystalline arrangement of PAN chains in two dimensions (Figure 9). The peaks, corresponding to the diffraction in the orthorhombic lattice, can be indexed as (110) and (200), respectively. The diffractogram of rGO nanoparticles (Figure 9) contains a broad peak at 2θ = 24°, which is associated with the agglomeration of individual graphene layers.

**Figure 10:**
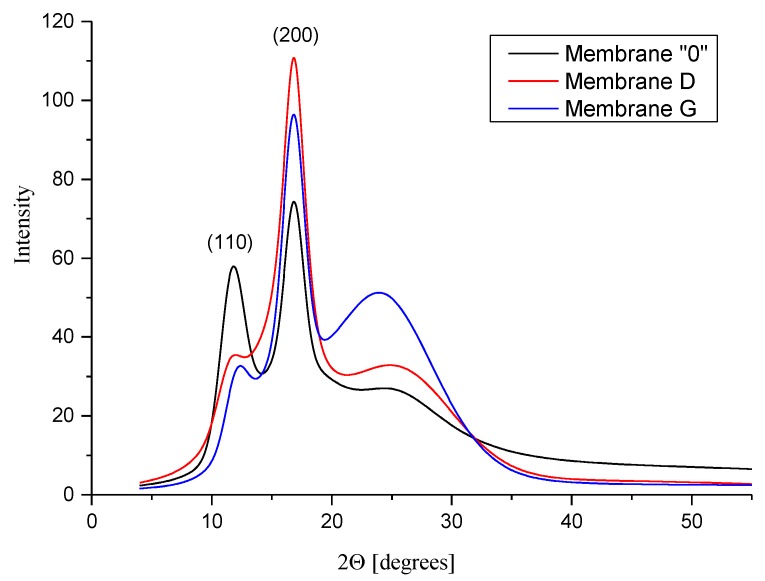
Comparison of X-ray diffractograms of membranes containing different rGO content.

The X-ray diffractograms of nanocomposite membranes (Figure 10) still exhibit the two main PAN peaks, however, their intensity evidently changes with the addition of graphene nanoparticles. The intensity of the peak (110) significantly decreases while intensity of the peak (200) grows. Moreover, the amorphous halo increases with increasing rGO content due to both the addition of amorphous rGO nanoparticles, and the increase of the PAN amorphous region content. This, in turn, is associated with a decrease in the crystallinity index of PAN with increasing rGO content in the membranes tested. Quantitative analysis of the diffractograms shows that for the rGO content greater than 1%, the crystallinity of the membranes decreases from 29.5% to 17.3% for membranes D and membranes G, respectively. These observations suggest that the addition of rGO particles changes the conditions under which polyacrylonitrile chains are ordered.

It has been recognized in many investigations that polymer crystallization is strongly influenced by the presence of additives because the nanometric size of the fillers leads to the increase of the interfacial area, thus creating a significant volume fraction of “interfacial” polymer chains which may crystallize under confinement [37]. Two kinds of nano-confinement effects on crystallization can be identified. In some cases, the attraction between the polymer chains and the surfaces of nanofillers leads to formation interfacial layer with reduced chain mobility, which exhibits different crystallization behavior than that of bulk polymer [38]. In other cases, confinement is introduced when the chains are restricted within the space between the nanoparticles. It should be emphasized that in addition to the size and shape of the nanoparticles, a significant effect on confinement crystallization has the filler loading and the dispersion of the nanoparticles within the polymer matrix. The higher the particle loading the smaller the distance between neighboring particles, which leads to increasing steric hindrance in the crystallization process.

#### 3.5.3. DSC

**Figure 11:**
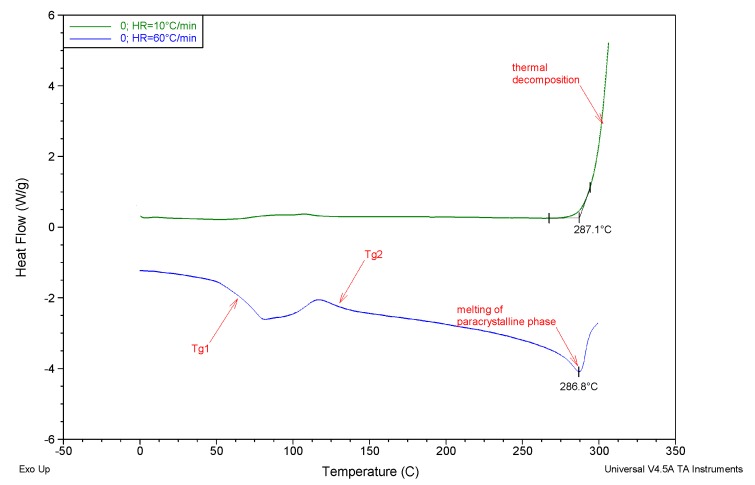
DSC curves for samples of unmodified PAN membrane (0) registered in the heating mode in nitrogen atmosphere with the rate of: 10°/min (green) and 60°/min (blue), respectively.

Figure 11 summarizes the DSC curves for samples of pure PAN membrane (membrane 0) recorded during heating at significantly differing rates. The curve corresponding to the rate of 10°/min, most common in conventional DSC measurements, clearly illustrates only the beginning of the PAN thermal decomposition process, well-described in the literature and consisting, in the first step, in the cyclization of the polymer chains. Furthermore, in the temperature range 60–140 °C on the discussed DSC curve there is a disruption of the calorimeter signal line which, is associated with the glass transition within the disordered PAN phase. However, the low intensity of the effect makes it practically impossible to be interpreted any deeper. With an increase of the temperature change dynamics up to 60 °/min, as a function of measurement time, complex thermal effects in terms of glass transition temperatures and a pronounced melting peak of paracrystalline PAN phase, preceding the onset of the polymer thermal decomposition, are reflected on the curves.

**Figure 12:**
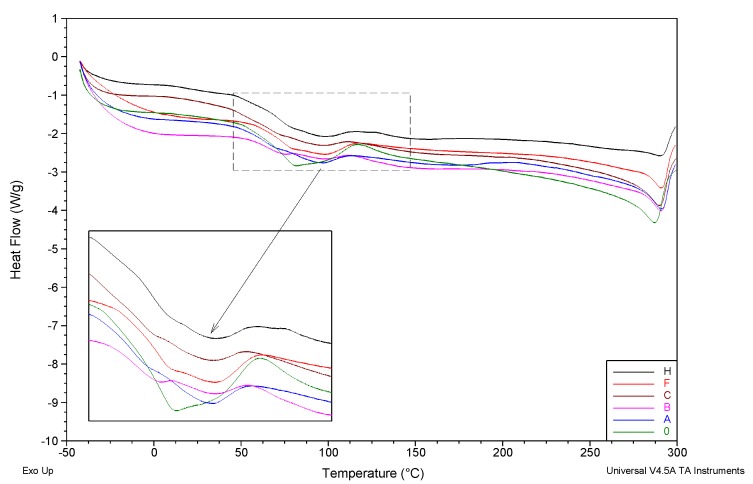
DSC curves for investigated membranes obtained from: unmodified PAN (green), and PAN modified with rGO (other colors). Curves registered in the heating rate of 60 °C/min in temperature ranges of: Glass transitions (of amorphous and mesomorphic phases), melting, and beginning of thermal decomposition, respectively.

The thermal effects mentioned in Figure 11 (marked on the curve marked with red arrows as *T_g_1* and *T_g_2*, respectively) are of a relaxing nature, and their occurrence is directly related to the transition from the thermodynamic glassy to viscoelastic state, within the amorphous PAN phase and mesomorphic phase, showing a certain type of ordering, referred to as: *glass-rubber* and *glass laterally-ordered*. In the case of membranes discussed in this paper, the first of the abovementioned transformations is located in the temperature range of 63.8–70.2 °C (Figure 12, Table 3), and the temperature shift of its characteristic *T_g_1* value does not show a clear tendency of change, as a function of increasing rGO content.

The second of the aforementioned relaxation transformations for the studied membranes is observed in the temperature range of 119.5–127.7 °C (Figure 12; Table 3) and also does not show a monotonic change trend. A consistent analysis of the recorded DSC curves, in the order of increasing temperature, leads to ultimate observation of the para-crystalline mesomorphic PAN phase melting effect (there is no ordering parallel to the axis of the chain). The minimum temperature of the melting peak *T_m_* is the lowest for membrane 0 (287.2 °C), and the highest (290.8 °C) for membrane B (modified with 0.0135% of rGO). For other membranes, *T_m_* adopts intermediate values with a trend decreasing with the increase in the rGO content. The divergence of *T_m_* value, which is usually correlated with the average size of crystallites, is small (though significant) within the whole series, and has a valve of 3.6 °C.

## 4. Conclusions

An innovative and simple method of obtaining composite membranes containing a wide range of concentrations of rGO (from 0.11% to 29.4%) in the PAN matrix was developed. The method enables good dispersion of the nano-additive in the polymer matrix, which was confirmed by structural studies. The addition of nanoparticles influenced also the physicochemical properties of the formed rGO/PAN membranes. Composite membranes were characterized by high porosity and hydrophilicity which resulted in very good transport properties. The obtained rGO/PAN membranes showed also good separation properties in relation to BSA. The addition of rGO in the PAN matrix effectively prevented the phenomenon of fouling, which may indicate a change in charge on the obtained membranes. An additional feature of rGO/PAN membranes are biostatic properties, confirmed by the study, preventing the development of bacteria and fungi on their surface, and thus the occurrence of adverse biofouling phenomenon. Biostatic properties combined with good transport and separation properties create, as Zhao et al. wrote [39], the potential possibility of their use in water disinfection processes. The undoubted advantage of the obtained composite membranes is the permanent fixing of rGO in the PAN matrix, which prevents the release of nanoparticles into the environment.

## Figures and Tables

**Table 1:** The composition of the solutions used for the membrane preparations.

Type of Membrane	Amount of rGO [g]	Amount of PAN [g]	Amount of DMF [g]	Conc. of rGO [% *w*/*w*]	Conc. of PAN [% *w*/*w*]
0	0	12.0	88.0	0	100.0
A	0.0135	12.0	87.9865	0.11	99.89
B	0.027	12.0	87.973	0.22	99.78
C	0.054	12.0	87.946	0.45	99.55
D	0.1	12.0	87.9	0.83	99.27
E	0.5	12.0	87.5	4.0	96.0
F	1.0	12.0	87.0	7.7	92.3
G	2.0	12.0	86.0	14.3	85.7
H	5.0	12.0	83.0	29.4	70.6

**Table 2:** Physicochemical properties of obtained membranes.

Membrane	l [µm]	W_s_ [g/cm^2^]	d_m_ [g/cm^3^]	U [%]	ε [%]	θ [deg]
0	190.1 ± 9.3	0.00322 ± 0.00021	0.170 ± 0.016	249.0 ± 20.6	74.5 ± 2.3	52.0 ± 2.8
A	198.8 ± 13.9	0.00300 ± 0.00019	0.151 ± 0.009	259.1 ± 18.4	74.6 ± 3.7	49.5 ± 2.7
B	188.9 ± 12.1	0.00300 ± 0.00023	0.159 ± 0.007	261.5 ± 19.9	75.1 ± 3.5	47.3 ± 4.1
C	182.5 ± 8.6	0.00264 ± 0.00017	0.145 ± 0.015	289.5 ± 25.5	76.9 ± 4.0	48.2 ± 2.7
D	182.8 ± 13.1	0.00256 ± 0.00014	0.140 ± 0.008	325.1 ± 20.9	77.0 ± 4.3	45.2 ± 2.6
E	170.7 ± 9.8	0.00246 ± 0.00019	0.144 ± 0.016	329.8 ± 23.5	79.4 ± 2.6	40.7 ± 3.3
F	169.6 ± 14.5	0.00252 ± 0.00017	0.149 ± 0.015	329.0 ± 18.3	79.1 ± 1.9	42.9 ± 4.3
G	168.8 ± 12.9	0.00258 ± 0.00023	0.153 ± 0.015	241.1 ± 25.5	78.8 ± 3.4	45.8 ± 4.0
H	167.6 ± 8.6	0.00290 ± 0.00017	0.173 ± 0.013	209.3 ± 24.4	75.3 ± 2.0	47.3 ± 4.6

where: l—membrane thickness; W_s_—mass per unit area; d_m_—apparent density; U—water sorption; ε—porosity; θ—contact angle.

**Table 3:** Values of characteristic temperatures of glass transition (appointed using half-height procedure) of amorphous T_g_1 and mesomorphic T_g_2 phases and melting T_m_, evaluated on the basis of DSC curves (Figure 2) for selected investigated PAN-rGO membranes.

Membrane	T_g_1 [°C]	T_g_2 [°C]	T_m_ [°C]
0	68.6	125.7	287.2
A	64.8	not visible	290.8
B	63.8	120.3	290.7
C	64.7	124.6	289.9
F	70.2	125.3	290.7
H	69.4	119.5	290.2

where: T_g_1—temperature of glass transition of amorhous phase; T_g_2—temperature of glass transition of mesomorphic phase, T_m_—temperature of melting.
